# Supplementary material for: Heat adaptation of phage T7 under an extended genetic code
Source: Virus Evol. 2021 Dec 1;7(2):veab100. doi: 10.1093/ve/veab100 (PMC8923235; doi:10.1093/ve/veab100)
Supplement: veab100_Supp [file veab100_supp.zip › Sequencing_Analysis.zip!/Sequencing_Analysis/A1/evidence/JC_SIDE_1_61.html]

BRESEQ :: Evidence


| New junction evidence | | | | | | | | | | | |
| --- | --- | --- | --- | --- | --- | --- | --- | --- | --- | --- | --- |
|  | | seq id | position | reads (cov) | reads (cov) | score | skew | freq | annotation | gene | product |
| \* | ? | NC\_001604.1 | = 13578 | 23 (0.010) | 22 (0.010) | 13/584 | 38.7 | 15.8% | intergenic (–/–) | *–/–* | –/– |
|  | ? | NC\_001604.1 | 14348 = | 212 (0.070) |  |  |  |  | intergenic (–/–) | *–/–* | –/– |
| Rejected: Coverage evenness skew score above cutoff. | | | | | | | | | | | |
| Rejected: Frequency below/above cutoff threshold. | | | | | | | | | | | |

Only 100 of 2359 total aligned reads displayed.  

`TGACTTTCCAGACGACTACGAGAAGTTTCGCTGGAGAGTCCCATTCTAATACGACTCACTAAAGGAGACACACCATGTTCAAACTGATTAAGAAGTTAGGCCAACTGCTGGTTCGTATGTACAACGTGGAAGCCAAGCGACTGAACGATGAGGCTCGTAAAGAGGCCACACAGTCACGCGCTCTGGCGATTCGCTCCAACGAACTGGCTGACAGTGCATCCACTAAAGTTACCGAGGCTGCCCGTGTGGCAAACCAAGCTCAACAGCTTTCCAAATTCTTTGAGTAATCAAACAGGAGAAACCATTATGTCTAACGTAGCTGAAACTATCCGTCTATCCGATACAGCTGACCAGTGGAACCGTCGAGTCCACATCAACGTTCGCAACGGTAAGGCGACTATGGTTTACCGCTGGAAGGACTCTAAGTCCTCTAAGAATCACACTCAGCGTATGACGTTGACAGATGAGCAAGCACTGCGTCTGGTCAATGCGCTTACCAAAGCTGCCGTGACAGCAATTCATGAAGCTGGTCGCGTCAATGAAGCTATGGCTATCCTCGACAAGATTGATAACTAAGAGTGGTATCCTCAAGGTCGCCA  >  NC_001604.1/13278‑13876`  

                                                                                                                                                                                                                                                                                                            `|`                                                                                                                                                                                                                                                                                                            
   `cTTTCCAGACGACTACGAGAAGTTTCGCTGGAGAGTCCCATTCTAATACGACTCACTAAAGGAGACACACCATGTTCAAACTGATTAAGAAGTTAGGCCAACTGCTGGTTCGTATGTACAACGTGGAAGCCAAGCGACTGAACGATGAGGCTCGTAAAGAGGCCACACAGTCACGCGCTCTGGCGATTCGCTCCAAAGAAATGGCTGACAGTGCATCCACTAAAGTTACCGATGCTGCCCGTGTGGCAAACCAAGCTCAACAGCTTTCCAAATTCTTTGAGTAATCAAACAGGAGAAACCa                                                                                                                                                                                                                                                                                                         >  1:369021/1‑301 (MQ=255)`  
          `gggctcggagatgtgtataagagacaggtcccattctaatacgactcactaaaggAGACACACCATGTTCAAACTGATTAAGAAGTTAGGCCAACTGCTGGTTCGTATGTACAACGTGGAAGCCAAGCGACTGAACGATGAGGCTCGTAAAGAGGCCACACAGTCACGCGCTCTGGCGATTCGCTCCAACGAACTGGCTGACAGTGCATCCACTAAAGTTACCGAGGCTGCCCGTGTGGCAAACCAAGCTCAACAGCTTTCCAAATTCTTTGAGTAATCAAACAGGAGAAACCATTATGat                                                                                                                                                                                                                                                                                                  <  1:4285‑M1/274‑3 (MQ=255)`  
              `acTACGAGAAGTTTCGCTGGAGAGTCCCATTCTAATACGACTCACTAAAGGAGACACACCATGTTCAAACTGATTAAGAAGTTAGGCCAACTGCTGGTTCGTATGTACAACGTGGAAGCCAAGCGACTGAACGATGAAGCTCGTAAAGAGGCCACACAGTCACGCGCTCTGGCGATTCGCTCCAACGAACTGGCTGACAGTGCATCCACTAAAGTTACCGAGGCTGCCCGTGTGGCAAACCAAGCTCAACAGCTTTCCAAATTCTTTGAGTAATCAAACAGGAGAAACCATTATGatcctg                                                                                                                                                                                                                                                                                              >  1:233406‑M1/1‑295 (MQ=255)`  
                           `tCGCTGGAGAGTCCCATTCTAATACGACTCACTAAAGGAGACACACCATGTTCAAACTGATTAAGAAGTTAGGCCAACTGCTGGTTCGTATGTACAACGTGGAAGCCAAGCGACTGAACGATGAGGCTCGTAAAGAGGCTACACAGTCACGCGCTCTGGCGATTCGCTCCAACGAACTGGCTGACAGTGCATCCACTAAAGTTACCGAGGCTGCCCGTGTGGCAAACCAAGCTCAACAGCTTTCCAAATTCTTTGAGTAATCAAACAGTAGAAACCATTATGatcgtttctgacatcgaag                                                                                                                                                                                                                                                                                 >  1:133414‑M1/1‑282 (MQ=255)`  
                           `tCGCTGGAGAGTCCCATTCTAATACGACTCACTAAAGGAGACACACCATGTTCAAACTGATTAAGAAGTTAGGCCAACTGCTGGTTCGTATGTACAACGTGGAAGCCAAGCGACTGAACGATGAGGCTCGTAAAGAGACCACACAGTCACGCACTCTGGCGATTCGCTCCAACGAACTGGCTGACAGTGCATCCACTAACGTTACCGAGGCTGCCCGTGTGGCAAACCAAGCTCAACAGCTTTCCAAATTCTTTGAGTAATCAAACAGGAGAAACCATTATGatcgtttctgacatcgaag                                                                                                                                                                                                                                                                                 >  1:230416‑M1/1‑282 (MQ=255)`  
                                    `gagacagttctaatacGACTCACTAAAGGAGACACACCATGTTCAAACTGATTAAGAAGTTAGGCCAACTGCTGGATCGTATGTACAACGTGGAAGCCAAGCGACTGAACGATGAGGCTCGTAAAGAGGCCACACAGTCACGCGCTCTGGCGATTCGCTCCAACGAACTGGCTTACAGTGCATCCACTAAAGTTACCGAGGCTGCCCGTGTGGCAAACCAAGCTCAACAGCTTTCCAAATTCTTTGAGTAATCAAACAGGAGAAACCATTATGatcgtttctgacatcgaagctaacgccc                                                                                                                                                                                                                                                                        <  1:392860‑M1/294‑29 (MQ=255)`  
                                     `gTCCCATTCTAATACGACTCACTAAAGTAGACACACCATGTTCAAACTTATTACAAAGTTAGGCCAACTGCTGGTTCGTATGTACAACGTGGAAGCCAAGCGACTGAACGATGAGGCTCGTAAAGAGGCCACACAGTCACGCGCTCTGACGATTCGCTCCAACGACCTGGCTGACAGTGCATCCACTAAAGTTACCGAGGCTGCCCGTGTGGCAAACCAAGCTCAACAGCTTTCCAAATTCTTTGAGTAATCAAATCGGAGAAACCATTATGaccgtttctgacatcgaagctaacgccct                                                                                                                                                                                                                                                                       >  1:307439‑M1/1‑272 (MQ=255)`  
                                     `gTCCCATTCTAATACGACTCACTAAAGGAGACACACCATGTTCAAACTGATTAAGAAGTTAGGCCAACTGCTGGTTCGTATGTACAACGTGGAAGCCAAGCGACTGAACGATGAGGCTCGTAAAGAGGCCACACAGTCACGCGCTCTGGCGATTCGCTCCAACGAACTGGCTGACAGTGCATCCACTAAAGTTACCGAGGCTGCCCGTGTGGCAAACCAAGCTCAACAGCTTTCCAAATTCTTTGAGTAATCAAACAGGAGAAACCATTATGatagtttctgacatcgaagctaacgcccc                                                                                                                                                                                                                                                                       >  1:220951‑M1/1‑272 (MQ=255)`  
                                     `gTCCCATTCTAATACGACTCACTAAAGGAGACACACCATGTTCAAACTGATTAAGAAGTTAGGCCAACTGCTGGTTCGTATGTACAACGTGGAAGCCAAGCGACTGAACGATGAGGCTCGTAAAGAGGCAACACAGTCACGCGCTCTGGCGATTCGCTCCAACGAACTGGCTGACAGTGCATCCACTAAAGTTACCGAGGCTGCCCGTGGGGCAAACCAAGCCCAACAGCTTTCCAAATTCTTTGAGTAATCAAACAGGAGAAACCATTATGatcggttctgacatcgaagctaacgccct                                                                                                                                                                                                                                                                       >  1:85717‑M1/1‑272 (MQ=255)`  
                                          `aTTCTAATACGACTCACTAAAGGAGACACACCATGTTCAAACTGATTAAGAAGTTAGGCCAACTGCTGGTTCGTATGTACAACGTGGAAGCCAAGCGACTGAACGATGAGGCTCGTAAAGAGGCCACACAGTCACGCGCTCTGGCGATTTGCTCCAACGAAATGGCTGACAGTGCATCCACTAAAGTTACCCAGGCTGCCCGTGTGGCAAACCAAGCTCAACAGCTTTCCAAATTCTTTGAGTAATCAAACAGGAGAAACCATCATGatagtttctgaaatcgaagctaacgccctcttag                                                                                                                                                                                                                                                                  >  1:16130‑M1/1‑267 (MQ=255)`  
                                           `ttCTAATACGACTCACTAAAGGAGACACACCATGTTCAAACTGATTAAGAAGTTAGGCCAACTGCTGGTTCGTATGTACAACGTGGAAGCCAAGCGACTGAACGATGAGGCTCGTAAAGAGGCCACACAGTCACGCGCTCTGTCGCTTCGCTCCAACGAACTGGCTGACAGTGCATCCACTAAAGTTACCGAGGCTGCCCGTGTGGCAAACAAAGCTCAACAGCTTTCCAAATTCTTTGAGTAATCCAACAGGAGAAACCATTATGatcgtttctgacatcgaagctaacgccctcttcga                                                                                                                                                                                                                                                                 >  1:469765‑M1/1‑266 (MQ=255)`  
                                           `ttCTAATACGACTCACTAAAGGAGACACACCATGTTCAAACTGATTAAGAAGTTAGGCCAACTGCTGGTTCGTATGTACAACGTGGAAGCCAAGCGACTGAACGATGAGGCTCGTAAAGAGGCCACACAGTAACGCGCTCTGGCGATTCGCTCCAACGAACTGGCTGACAGTGCATCCACTAAAGTTACCGAGGCTTCCCGTGTGGCAAACCAAGCTCAACAGCTTTCCAAATTCTTTTAGTAATCAAACAGGAGATACCATTATGatcgtttctgacatcgaagctaacgccctctttga                                                                                                                                                                                                                                                                 >  1:174306‑M1/1‑266 (MQ=255)`  
                                           `ttCTAATACGACTCACTAAAGGAGACACACCATGTTAAAACTGATTAAGAAGTTAGGCCAACTGCTGGTTCGTATGTACAACGTGGAAGCCAAGCGACTGAACGATGAGGCTCGTAAAGAGGCCACACAGTCACGCGCTCTGGCGATTCGCTCCAACGAACTGGCTGACAGTGCATCCACTAAAGTTACCGAGGCTGCCCGTGTGGCAAACCAAGCTCAACAGCTTTCAAAATTCTTTGAGTAATCAAACAGGAGAAACCATTATGatcgtttctgacatcgaagctaacgccctcttaga                                                                                                                                                                                                                                                                 >  1:409471‑M1/1‑266 (MQ=255)`  
                                            `tCTAATACGACTCACTAAAGGAGAAAAACCATGTTCAAACTGATTAAGAAGTTAGGCCAAATGCTGGTTCGTATGTACAACGTGGAAGCCAAGCGACTGAACGATGAGGCTCGTAAAGAGGCCACACAGTCAAGCGCTCTGGCGATTCGCTCCAACGAACTGGCTGACAGTGCATCCACTAAAGTTACCGAGGCTGCCCGTGTGGCAAACCAAGCTCAACAGCTTTCCAAATTCTTTGAGTAATCAAACAGGAGAAACCATTATGatagtttctgacatcgaagctaacgccctcttagag                                                                                                                                                                                                                                                                <  1:294371‑M1/301‑37 (MQ=255)`  
                                            `gctAATACGACTTACTAAAGTATACACACCATGTTCAAACTGATTAAGAAGTTAGGCCAACTGCTGGTTCGTATGTACAACGTGGAAGCAAAGCGACTGAACGATGAGGCTCGTAAAGAGGCCACACAGTCACGCGCTCTGGCGATTCGCTCCAACGAACTGGCTGACAGTGCATCCACTAAAGTTACCGAGGCTGGCCGTGTGGCAAACCAAGCTCAACAGCTTTCCAAATTCTTTGAGTAATCAAACAGGAGAAACCATTATGatcgtttctgacatcgaagctaacgccctcttagag                                                                                                                                                                                                                                                                <  1:45396‑M1/300‑37 (MQ=255)`  
                                            `agatgtgtataagagacagggagacacaccatgttcaaacTGATTAAGAAGTTAGGCCAACTGCTGGTTCGTATGTACAACGTGGAAGCCAAGCGACTGAACGATGAGGCTCGTAAAGAGGCCACACAGTCACGCGCTCTGGCGATTCGCTCCAACGAACTGGCTGACAGTGCATCCACTAAAGTTACCGAGGCTGCCCGTGTGGCAAACCAAGCTCAACAGCTTTCCAAATTCTTTGAGTAATCAAACAGGAGAAACCATTATGatcgtttctgacatcgaagctaacgccctcttagag                                                                                                                                                                                                                                                                <  1:156501‑M1/282‑37 (MQ=255)`  
                                              `tAATACGACTCACTATAGGAGACACACCATGTTCAAACTGATTAAGAAGTTAGGCCAACTGCTGGTTCGTATGTACAACGTGGAAGCCAAGCTACTGAACGATGAGGCTCGTAAAGAGGCCACACAGTCACGCGCTCTGGCGATTCGCTCCAACGAACTGGCTGACAGTGCATCCACTAAAGCTACCGAGGCTGCCCGTGTGGCAAACCAAGCTCAACAGCTTTCTAAAATCTTTGAGTAATCAAACAGGAGAAACCATTATGatcgtttctgatatagaagctaaagccctcttagagag                                                                                                                                                                                                                                                              >  1:106304‑M1/1‑263 (MQ=255)`  
                                                `aTACGACTCACTAAAGGAGACACACCATGTTCAAACTGATTAAGAAGTTAGGCCAACTGCTGGTTCGTATGTACAACGTGGAAGCCAAGCGACTGAACGATGAGGCTCGTAAAGAGGCCACACAGTCACGCGCTCTGGCGATTCGCTCCAACGAACTGGCTGACAGTGCATCCACTAAAGTTACCGACGCTGCCCGTGTGGCAAACCAAGCTCAACAGCTTTCCAAATTCTTTGAGTAATCAAACAGGAGAAACCATTAGGatcgtttctgacatcgaagcctgtctcttatacacatctc                                                                                                                                                                                                                                                            >  1:348256‑M1/1‑261 (MQ=255)`  
                                                        `cACTAAAGGAGACACACCATGTTCAAACTGATTAAGAAGTTAGGCCAACTGCTGGTTCGTATGTACAACGTGGAAGCCAAGCGACTGAACGATGAGGCTCGTAAAGAGGCCACACAGTCACGCGCTCTGGCGATTCGCTCCAACGAACTGGCTGACAGTGCATCCACTAAAGTTACCGAGGCTGCCCGTGTGGCAAACCAAGCTCAACAACTTTCCAAATTCTTTGAGTAATCAAACAGGAGAAACCATTATGatcgtttctgacatcgaagctaacgccatcttagaaagcgtcactaag                                                                                                                                                                                                                                                    >  1:441121‑M1/1‑253 (MQ=255)`  
                                                         `aCTAAAGGAGACACACCATGTTCAAACTGATTAAGAAGTTAGGCCAACTGCTGGTTCGTATGTACAACGTGGAAGCCAAGCGACTGAACGATGAGGCTCGTAAAGAGGCCACACAGTCACGCGCTCTGGCGATTCGCTCCAACGAACTGGCTGACAGTGCATCCACTAAAGTTACCGAGGCTGCCCGTGTGGCAAACCAAGCTCAACAGCTTTCCAAATTCTTTGAGTAATCAAACAGGAGAAACCATTATGatcgtttctgacatcgaagctaaagccctcttagagagcgtcattaagt                                                                                                                                                                                                                                                   >  1:47387‑M1/1‑252 (MQ=255)`  
                                                           `tgtgtataagagacagtgttcaaactgagtaagAAGTTAGGCCAACTGCTGGTTCGGATGTACAACGTGGAAGCCAAGCGACTGAACGATGAGGCTCGTAAAGAGGCCACACAGTCACGCGCTCTGGCGATTCGCTCCAACGAACTGGCTGACAGTGCATCCACTAAAGTTAACGAGGCTGCCCGTGTGGCAAACCAAGCTCAACAGCTTTCCAAATTCTTTGAGTAATCAAACAGGAGAAACCATTATGatcgtttctgacatcgaagctaacgccctcttagagagcgtcactaagttc                                                                                                                                                                                                                                                 <  1:504082‑M1/285‑52 (MQ=255)`  
                                                              `aGGAGACACACCATGTTGAAACTGATTAGGAATTTAGGCCAACGGCAAGTTCGTATGTACAGCGTGAAAGCCAAGCGACTGAACGATGAGGCTCGTAAAGAGGCCACACAGTCACGCGCTCTGGCGATTCGCTCCAACGAACTGGCTGACAGTGCATCCACTAAAGTTAGCTAGTCTGCCCGTGTGGCAAACCAAGCTCAACAGCTTTCAAAATTCTTTGAGTAATCCAACAGGAGAAACCATTATGatcgtttctgacatcgaagctaacgccctcttagagagcgtcactaagttccac                                                                                                                                                                                                                                              <  1:2248‑M1/301‑55 (MQ=255)`  
                                                               `ggAGACACACCATGTTCAAACTGATTAAGAAGTTAGGCCAACTGCTGGTTCGTATGTAAAACGTGGAAGCCAAGCGACTGAACGATGAGGCTCGTAAAGAGGCCACCCAGTCACGCGCCCTGGCCATTCGCTCCAACGAACTGGCTGACAGTGCATCCACTAAAGTTACCGAGCCTGCCTGTGTGGCAAACCAAGCTCAACAGCTTTCCAACTTCTTTGAGTAATCAAACAGGAGAAACCATCATGatcgtttctgacatacaagctaaagacatcttagcgagcgtcacgaagtgcctgc                                                                                                                                                                                                                                             >  1:146580‑M1/1‑246 (MQ=255)`  
                                                                 `agaCACACCATGTTCAAACTGATTAAGAAGTTAGGCCAACTGCTGGTTCGTATGTACAACGTGGAAGCCAAGCGACTGAACGATGAGGCTCGTAAGGAGGCCACACAGTCACGCGCTCTGGCGATTCGCTCCAACGACCTGGCTGACAGTGCATCCACTAAAGTTACCGAGGCTGCCCGTGTGGCAAACCAAGCTCAACAGCTTTCCAAATTCTTTGAGTAATCAACCAGGAGAAACCATTATGatcgtttctgacatcgaagctaacgccctcttagtgagcgtcactacgttccactgc                                                                                                                                                                                                                                           >  1:237672‑M1/1‑244 (MQ=255)`  
                                                                   `atagacagaggtcatactgattaAGAAGTGAGGTCAACTGCTTGTTCGTATGTACATCTTGGAAGCCAAGCGACTGAACGATGAGGCTCTGAAAGATGCTACACAGGCACGCTCTCTGGCGATTCGCATCAACGAACTGGTGGACAGTGCATCCACTAAAGTTACCGAGGCTGCCCGTGTGGCAAACCAAGCTCAACAGCTTTCCTAATTCTTTGAGTATTCAAACAGGAGAAATCATTATGatcgtttctgacatcgaagctaacgccctcttagagagcgtcactaagttccactgcgg                                                                                                                                                                                                                                         <  1:259627‑M1/290‑60 (MQ=255)`  
                                                                           `tGTTCAAACTGATTAAGAAGTTAGGCCATCTGCTGGTTCGTATGTACAACGTGGAAGCCAAGCGACTGAACGATGAGGCTCGTAAAGAGGCCACACAGTCACGCGCTCTGGCGATTCGCTCCAACGAACTGGCTGACAGTGCATCCACTAAAGTTACCGAGGCTGCCCGTGTGGCAAACCAAGCTCAACAGCTTTCCAAATTCTTTGAGTAATCAAACAGGAGAAACCATTATGatcgtttctgacatcgaagctaacgccctcttagagagcgtcactaagttccactgcggggttatct                                                                                                                                                                                                                                 <  1:387582‑M1/301‑68 (MQ=255)`  
                                                                           `tGTTCAAACTGATTAAGAAGTTAGGCCAACTGCTGGTTCGTATGTACAACGTGGAAGCCAAGCGACTGAACGATGAGGCTCGTAAAGAGGCCACACAGTCACGCGCTCTGGCGATTCGCTCCAACGAACTGGCTGACAGTGCATCCACTAAAGTTACCGAGGCTTCCCGTGTGGCAAACCAAGCTCAACAGCTTTCCAAATTCTTTGAGTAATC‑AATAGGAGAAATCAATATGatcgtttctgacatcgaagctaacgccctcttagagagcgtcactaagttccactgcggggttatcta                                                                                                                                                                                                                                >  1:143111‑M1/1‑233 (MQ=255)`  
                                                                            `gTTCAAACTGATTAAGAAGTTAGGCCAACTGCTGGTTCGTATGTACAACGTGGAAGCCAAGCGACTGAACGATGAGTCTCGTAAAGAGGCCACACAGTCACGCGCTCTGGCGATTCGCTCCAACGAACTGGCTGACAGTGCATCCACTAAAGTTACCGAGGCTGCCCGTGTGGCAAACCAAGCTCAACAGCTTTCCAAATTCTTTGAGTAATCAAACAGGAGAAACCATTATGatcgtttctgacatcgaagctaacgccctcttagagagcgtcactaagttccactgcggggttatcta                                                                                                                                                                                                                                >  1:184168‑M1/1‑233 (MQ=255)`  
                                                                                `aaaCTGATTAATGAGTTAGGCAAACTGATGGTTCGGATGTACAACGTGGAAGCCAAGCGACGGAACGATGAGGCTCGTAAAGAGGCCACACAGTCACGCGCTGTGGCGATTAGATCCAACGAACTGGCTGACAGTGCATCCACTAAAGTTACCGAGGCTGCCCGTGTGGCAAACCAAGCTCAACAGCTTTCCAAATTCTTTGAGTAATCAAACAGGAGAAACCAATATGatcgtttctgacatcgaagctaacgccctcttagagagcgtcactaagttccactgcggggttatctacgac                                                                                                                                                                                                                            <  1:5271‑M1/301‑73 (MQ=255)`  
                                                                                `aaaCTGATTAAGAATTTAGGCCAACTGCTGGTTCGTATGTACAACGTGGAAGCCAAGCTACTGAACGATGAGGCTCGTAAAGAGGCCACACAGTCACGCGCTCTGGCGATTCGCTCCAACGAACTGGCTGACAGTGCATCCACTAAAGTTACCGAGGCTGCCCGTGTGGCAAACCAAGCTCAACAGCTTTCCAAATTCTTTGAGTAATCAAACAGGAGAAACCATTATGatcgtttctgacatcgaagctaacgccctcttagagagcgtcactaagttccactgcggggttatctacgac                                                                                                                                                                                                                            <  1:116712‑M1/301‑73 (MQ=255)`  
                                                                                `aaaCTGATTAAGAAGTTAGGCCAACTGCTGGTTCGTATGTACAACGTGGAAGCCAAGCGACTGAACGATGAGGCTCGTAAAGAGGCCACACAGTCACGCGCTCTGGCGATTCGCTCCAACGAACTGGCTGACAGTGCATCCACTAAAGTTACCGAGGCTGCCCGTGTGGCAAACCAAGCTCAACAGCTTTCCAAATTCTTTGAGTAATCAAACAGGAGAAACCATTATGatcgtttctgacatcgaagctaacgccctcttagagagcgtcactaagttccactgcggggttatctacgac                                                                                                                                                                                                                            <  1:465093‑M1/301‑73 (MQ=255)`  
                                                                                   `cTGATTAAGAAGTTAGGCCAACTGCTGGTTCGTATGTACAACGTGGAAGCCAAGCGACTGAACGATGAGGCTCGTAAAGAGGCCACACAGTCACGCGCTCTGGCGATTCGCTCCAACGAACTGGCTGACAGTGCATCCACTAATGTTACCGAAGCTGCCCGTGTGGCAAACCACGCTCAACAGCTTTCCAAATTCTTTGAGTAATCAAACAGGAGAAACAATTATGatcgtatcttacatcgaagctaacgccctcttagatagcgcccctaagttcccctgcggggttatctacgactac                                                                                                                                                                                                                         >  1:80947‑M1/1‑226 (MQ=255)`  
                                                                                     `gATTAAGAAGTTAGGCCAACTGCTGGTTCGTATGTACAACGTGGAAGCCAAGCGACTGAACGATGAGGCTCGTAAAGAGGCCACACAGTCACGCGCTCTGGCGATTCGCTCCAACGAACTGGCTGACAGTGCATCCACTAAAGTTACCGAGGCTGCCCGTGTGGCAAACCAAGCTCAACAGCTTTCCAAATTCTTTGAGTAATCAAACAGGAGAAACCATTATGatcgtttctgacatcgaagctaacgccctcttagagagcgtcactaagttccactgcggggttatctacgactactc                                                                                                                                                                                                                       >  1:151971‑M1/1‑224 (MQ=255)`  
                                                                                         `aagaagGTAGGCCAACTGCTGGTTCGAATGAACAACGTAGAAGCCAAGCGACTGAACGATGAGGCTAGTAAAGAGGCCACACAGTCACGCGCTCTGGCGATTCGCTCCAACGAACTGGCTGACAGTGCATCCACTAAAGTTACCGAGGCTGCCCGTGTGGCAAACCAAGCTCAACAGCTTTCAAAATTCTTGGAGTAATCAAACAGGAGAAATAAATATGatcgtttctgacatcgaagctaacgccctcttagagagcgtcactaagttccactgcggggttatctacgactactccacc                                                                                                                                                                                                                   <  1:344273‑M1/301‑82 (MQ=255)`  
                                                                                            `aagTTAGGCCAACTGCTGGTTCGTATGTACAACGTGGAAGCCAAGCGACTGAACGATGAGGCTCGTAAAGAGGCCACACAGTCACTCGCTCTGGCGATTCGCTCCAACGAACTGGCTGACAGTGCATCCACTAAAGTTACCGAGGCTGCCCGTGTGGCAAACCAAGCTCAACAGCTTTCCAAATTCTTTGAGTAATCAAACAGGAGAAACCATTATGatcgtttctgacatcgaagctaacgccctcttagagagcgtcactaagttccactgcggggttatctacgactactccaccgct                                                                                                                                                                                                                >  1:304316‑M1/1‑217 (MQ=255)`  
                                                                                            `aagTTAGGCCAACTGCTGGTTCGTATGTACAACGTGGAAGCCAAGCGACTGAACGATGAGGCTCGTAAAGAGGCCACACAGTCACGCGCTCTGGCGATTCGCTCCAACGAACTGGCTGACAGTGCATCCACTAAAGTTACCGAGGCTGCCCGTGGGGCAAACCAAGCTCAACAGCTTTCCAAATTCTTTGAGTAATCAAACAGGAGAAACCATTATGatcgtttctgacatcgaagctaacgccctcttagagagcgtcactaagttccactgcggggttatctacgactctgtctcttat                                                                                                                                                                                                                >  1:272067‑M1/1‑217 (MQ=255)`  
                                                                                             `agTTAGGCCAACTGCTGGTTCGTATGTACAACGTGGAAGCCAAGCGACTGAACGATGAGGCTCGTAAAGAGGCCACACAGTCACGCGCTCTGGCGATTCGCTCCAACGAACTGGCTGACAGTGCATCCACTAAAGTTACCGAGGCTGCCCGTGTGGCAAACCAAGCTCAACAGCTTGCCAAATTCTTTGAGTAATCAAACAGGATAAACCATTATGatcgtttctgacatcgaagctaacgccctcttatatctcgtcactaagttccactgctgggttatctacgtcctgtctcttataa                                                                                                                                                                                                               >  1:414508‑M1/1‑216 (MQ=255)`  
                                                                                                   `ggctcggagatgtttataagagacaggtggaagccaagcgactgaacgatgagGCTCGTAAAGAGGCCACACAGTCACGCGCTCTGGCGATTCGCTCCCACGAACTGGCTGACAGTGCATCCACTAAAGTTACCGAGGCTGCCCGTGTGGCAAACCAAGCTCAACAGCTTTCCAAATTCTTTGAGTAATCAAACAGGAGAAACCATTATGatcgtttctgacatcgcagctaacgccctcttagagagcgtcactaagttccactgcggggttatctacgactactccaccgctgagtacg                                                                                                                                                                                                         <  1:193599‑M1/275‑92 (MQ=255)`  
                                                                                                   `gCCAACTGCTGGTTCGTATGTACAACGTGGAAGCCAAGCGACTGAACGATGAGGCTCGTAAAGAGGCCACACAGTCACGCGCTCTGGCGATTCGCTCCAACGAACTGGCTGACAGTGCATCCACTAAAGTTACCGAGGCTGCCCGTGTGGCAAACCAAGCTCAACAGCTTTCCAAATTCTTTGAGTAATCAAACAGGAGAAACCATTATGatcgtttctgacatcgaagctaacgccctcttagagagcgtcactaagttccactgcggggttatctacgactactgtctcttatccacat                                                                                                                                                                                                         >  1:444406‑M1/1‑210 (MQ=255)`  
                                                                                                       `actgcgtgtgtctagttaccacgggtaagccaaGCGACTGATCGATGAGTCTCGTAAAGAGTCCACACAGTCACGCGCTCTGGCGATTCGCTCCATCGAACTGGCTGACAGTGCATCCACTAAAGTTACCGAGGCTGCCCGGGTGGCAAACCAAGCTCAACAGCTTTACAAATTCTTTGAGTAATCAAACAGTAGAAACCATTATGatcgtttctgccatcgaagctaacgccctcttagatagcgtcactaagttccactgcggggttatctactactactccaccgctgagtacataag                                                                                                                                                                                                     <  1:77728‑M1/285‑96 (MQ=255)`  
                                                                                                        `ctgctgGTTCGTATGTACAACGTGGAAGCCAAGCGACTGAACGATGAGGCTCGTAAAGAGGCCACACAGTCACGCGCTCTGGCGATTCGCTCCAACGAACTGGCTGACAGTGCATCCACTAAAGTTACCGAGGCTGCCCGTGTGGCAAACCAAGCTCAACAGCTTTCCAAATTCTTTGAGTAATCAAACAGGAGAAACCATTATGatcgtttctgacatcgaagctaacgccctcttagagagcgtcactaagttccactgcggggttatctacgactactccactgctgagtacgtaagc                                                                                                                                                                                                    >  1:61706‑M1/1‑205 (MQ=255)`  
                                                                                                               `ttCGTATGTACAAAGTTGAAGCCCAGCGCCTGAACGTTGAGGCTCGTAAAGAGGCCACACAGTCCCGCGCTCTGGCGATTCGCTCCAACGAACTGGCTGACAGTGCATCCACTAAAGTTACCGAGGCTGCCCGTGTGGCAAACCAAGCTCAACAGCTTTCCAAATTCTTTGAGTAATCAAACAGGAGAATCCATTATGatcgtttctgacatcgaagctaacgccctcttagagagcgtcactaagttccactgcggggttatctacgactactccaccgctgagtacgtaagctaccgtc                                                                                                                                                                                             <  1:508889‑M1/301‑104 (MQ=255)`  
                                                                                                                 `cGTATGTACAACGTGGAAGCCAAGCGACTGAACGATGATGCTCGTAAAGAGGACACACAGTCACGCGCTCTGGCGATTCGCTCCAACGAACTGGCTGACAGTGCCTCCACTAAAGTTACCGAGGCTTCCCGTGTTGCAAACCAAGCTCAACAGCTTTCCAAATTCTTTGAGTAATAAAACAGGAGAAACCATTATGatcgtttctgacatcgaagataacgccctcttagagatcgtcactaagttccacttcggggttatctacgaatactccaccgctgagtacctgtctcttatacaa                                                                                                                                                                                           >  1:339340‑M1/1‑196 (MQ=255)`  
                                                                                                                 `cGTATGTACAACGTGGAAGCCAAGCGACTGAACGATGAGGCTCGTAAAGAGGCCACACAGTCACGCGCTCTGGCGATTCGCTCCAACTAACTGGCTGACAGTGCATCCACTAAAGTTACCGAGTCTGCCCGTGTGGCAAACCAAGCTCAACAGCTTTCCAAATTCTTTGAGTAATCCAACAGGAGAAACCATTATGatcgtttctgacatcgaagctaactccctcttagagatcgtcactaagttccactgcggggttatctacgactactccaccgctgattacgtaagctaccgtccg                                                                                                                                                                                           >  1:357257‑M1/1‑196 (MQ=255)`  
                                                                                                                 `cGTATGTACAACGTGGAAGCCAAGCGACTGAACGATGAGGCTCGTAAAGAGGCCACACAGTCACGCGCTCTGGCGATTCGCTCCAACGAACTGGCTGACAGTGCATCCACTAAAGTTACCGAGGCTGCCCGTGTGGCAAACCAAGCTCAACAGCTTTCCAACTTCTTTGAGTAATCAAACAGGAGAAACCATTATGatcgtttctgacatcgaagctaacgccctcttagagggcgtcactaagttccactgcggggttatctacgactactccaccgctgcgtacgtaagctaccgtccc                                                                                                                                                                                           >  1:405785‑M1/1‑196 (MQ=255)`  
                                                                                                                 `cGTATGTACAACGTGGAAGCCAAGCGACTGAACGATGAGGCTCGTAAAGAGGCCACACAGTCACGCGCTCTGGCGATTCGCTCCAACGAACTGGCTGACAGTGCATCCACTAAAGTTACCGAGGCTGCCCGTGTGGCAAACCAAGCTCAACAGCTTTCCAAATTCTTTGAGTAATCAAACAGGAGAAACCATTATGatcgtttctgacatcgaagctaacgccctcttagagagcgtcactaagttccactgaggggttatctacgactactccaccgctgagtacgtaagctaccgtccg                                                                                                                                                                                           >  1:189245‑M1/1‑196 (MQ=255)`  
                                                                                                                 `cGTATGTACAACGTGGAAGCCAAGCGACTGAACGATGAGGCTCGTAAAGAGGCCACACAGTCACGCGCTCTGGCGATTCGCTCCAACGAACTAGCTGACAGTGCATCCACTAAAGTTACCGAGGCTGCCCGTGTGGCAAACCCAGCTCAACAGCTTTCCAAATTCTTTGAGTAATCAAACAGGAATACCCATTATGatcgtttctgacatctaaactaacgccctcttatagagcgtcactcagttctcctgcgggcttatttacttctactacaccgctcagtacttacgctaccgtccc                                                                                                                                                                                           >  1:498977‑M1/1‑196 (MQ=255)`  
                                                                                                                  `gTATGTACAACGTGGAAGCCAAGCGACTGAACGATGAGGCTCGTAAAGAGGCCACACAGTCACGCGCTCTGGCGATTCGCTCCAACGAACTGGCTGACAGTGCATCCACTAAAGTTACCGAGGCTGCCCGTGTGGCAAACCAAGCTCAACAGCTTTCCAAATTCTTTGAGTAATCAAACAGGAGAAACCATTATGatcgtttctgacatcgaagctaacgccctcttagagagcgtcactaagttccactgcggggttatctacgactactcaaccgctgagtacgtaagctggctcttct                                                                                                                                                                                          >  1:198365‑M1/1‑195 (MQ=255)`  
                                                                                                                     `tGTACAACGTGGAAGCCAAGCGACTGAACGATGAGGCTCGTAAAGAGGCCACACAGTCACGCGCTCTGGCGATTCGCTCCAACGAACTGGCTGACAGTGCATCCACTAAAGTTACCGAGGCTGCCCGTGTGGCAAACCAAGCTCAACAGCTTTCCAAATTCTTTGAGTAATCAAACAGGAGAAACCATTATGatcgtttctgacatcgaagctaacgccctcttagagagcgtcactaagttccactgcggggttatctacgactactccaccgctgagtacgtaagctacctgtctctta                                                                                                                                                                                       >  1:263304‑M1/1‑192 (MQ=255)`  
                                                                                                                        `aCAACGTGGAAGCCAAGCGACTGAACGATGAGGCTCGTAAAGAGGCCACACAGTCACGCGCTCTGGCGATTCGCTCCAACGAACTGGCTGACAGTGCATCCACTAAAGTTACCGAGGCTGCCCGTGTGGCAAACCAAGCTCAACAGCTTTCCAAATTCTTTGAGTAATCAAACAGGAGAAACCATTATGatcgtttctgacatcgaagctaacgccctcttagagagcgtcactaagttcaactgcggggttatctacgactactccaccgctgagtacgtaagctaccgtccgagtgact                                                                                                                                                                                    <  1:364633‑M1/301‑113 (MQ=255)`  
                                                                                                                           `aCGTGGAAGCCAAGCGACTGAACGATGAGGCTCGTAAAGAGGCCACACAGTCACGCGCTCTGGCGATTCGCTCCAACGAACTGGCTGACAGTGCATCCACTAAAGTTACCGAGGCTGCCCGTGTGGCAAACCAAGCTCAACAGCTTTCCAAATTCTTTGAGTAATCAAACAGGAGAAACCATTATGatcgtttctgacatggaagctaacgccctcttagagagcgtcactaagttctactgcggggttatctacgcctactccaccgctgagtacgtaagctaccgtccgagtgacttcg                                                                                                                                                                                 >  1:352905‑M1/1‑186 (MQ=255)`  
                                                                                                                            `cGTGGAAGCCAAGCGACTGAACGATGAGGCTCGTAAAGAGGCCACACGGTCACGCGCTCTGGCGATTCGCTCCAACGAACTGGCTGACAGTGCATCCACTAAAGTTACCGAGGCTGCCCGTGTGGCAAACCAAGCTCAACAGCTTTCCAAATTCTTTGAGTAATCAAACAGGAGAAACCATTATGatcgtttctgacatcgaagctaacgccctcttagagagcgtcactaagttccactgcggggttatctacgactactccaccgctgagtacgtaagctaccgtccgagtgacttcgg                                                                                                                                                                                >  1:285351‑M1/1‑185 (MQ=255)`  
                                                                                                                              `tGGAAGCCAAGCGACTGAACGATGAGGCTCGTAAAGAGGCCACACAGTCACGCGCTCTGGCGATTCGCTACAACGAACTGGATGACAGTGCATCCACTAAAGTTACCGAGGCTGCCCGTGTGGCAAACCAAGCTCAACAGCTTTCCAAATTCTTTGAGTAATCAAACAGGAGAAACCATTATGatcgtttctgacatcgaagctaacgccctcttagagagcgtcactaagttccactgcggggttatctacgactactccaccgctgagtacgtaagctaccgtccgagtgacttcggtg                                                                                                                                                                              <  1:214172‑M1/301‑119 (MQ=255)`  
                                                                                                                                    `ccAAGGGACTGAACGATGAGGCTCGGAAAGAGGCCACACAGTCACGCGCTCTGGCAATTCGCCACAACGAAATGGCTGAAAGGGCATCCAATAAAGTTACCGAGGCTGCCCGTGTGGCAAACCAAGCTAAAAAGCTTTCCAAATTCTTTGAGTAATAAAACAGGAGAAACCATTATGatcgtttctgacatcgaagctaacgcccgcttagagagagtcactaagttccactgcggggttatctacgactactccaccgctgagtacgtaagctaccgtccgagtgacttcggtgcgtatc                                                                                                                                                                        <  1:420155‑M1/301‑125 (MQ=255)`  
                                                                                                                                    `ccAAGCGACTGAACGATGAGGCTCGTAAAGAGGCCACACAGTCACGCGCTCTGGCGATTCGCTCCAACGAACTGGCTGACAGTGCATCCACTAAAGTTACCGAGGCTGCCCGTGTGGCAAACCAAGCTCAACAGCTTTCCAAATTCTTTGAGTAATCAAACAGGAGAAATCAATATGatcgtttctgacatcgaagctaacgccatcttagagagcgtcactaagttccactgcggggttatctactactactacaccgctgagtacgtaagctaccgtccgagtgccttcggtgcgtatc                                                                                                                                                                        >  1:328243‑M1/1‑177 (MQ=255)`  
                                                                                                                                        `gCGACTGAACGATGAGGCTAGTAAAGAGGCCACACAGTCACGCGCTCTGGCTATTCGCTCCAACGAACTGGTTGACAGTGCATCCACTAAAGTTACCGAGGCTGCCCGTGTGGCAAACCAAGCTCAACAGCTTTCCAAATTCTGTGAGTAATCAAACAGGAGAAATCAATATGatcgtttctgacatcgaagctaacgccctcttagagagcgtcactaagttccactgcggggttatctacgactactccaccgctgagtacgtaagctaccgtccgagtgacttcggtgcgtatctgga                                                                                                                                                                    <  1:56857‑M1/301‑129 (MQ=255)`  
                                                                                                                                        `gCGACTGAACGATGAGGCCCGTAAAGAGGCCACACAGTCACGCGCTCTGGCGATTCGCTCCAACGAACTGGCTGACAGTGCATCCACTAAAGTTACCGAGGCTGCCCGTGTGGCAAACCAAGCTCAACAGCTTTCCAAATTCTTTGAGTAATCAAACAGGAGAAACCATTATGatcgtttctgacatcgaagctaacgccctcttagagagcgtcactaagttccactgcggggttatctacgactactacaccgctgagtacgtaagctaccgtccgagtgacttcggtgcgtatctgga                                                                                                                                                                    <  1:121570‑M1/301‑129 (MQ=255)`  
                                                                                                                                        `gCGACTGAACGAGGAGGCTCGTAAAGAGGCCACACAGTCACGCGCTCGGGCGATTCGCTCCAACGAACTAGCTGACAGTGCATCCACTAAAGTTACCGAGGCTGCCCGTGTGGCAAACCAAGATCAACAGCTTTCCAAATTCTTTGAGTAATCAAACAGGAGAAACCATTATGatcgtttctgacatcgaagctaacgccctcttagagagcgtcactaagttccactgcggggttatctacgactactccaccgctgagtacgtaagctaccgtccgagtgacttcggtgcgtatctgga                                                                                                                                                                    <  1:243821‑M1/301‑129 (MQ=255)`  
                                                                                                                                                  `gagacagctcgtaAAGAGGCCACACAGTCACGCGCTCTGGCGATTCGCTCCAACGAACTGGCTGACAGTGCATCCACTAAAGTTACCGAGGCTGCCCGTGTGGCAAACCAAGCTCAACAGCTTTCCAAATTCTTTGAGTAATCAAACAGGAGAAACCATTATGatcgtctctgacatcgaagctaacgccctcttagagagcgtcactaagttccactgcggggttatctacgactactccaccgctgagtacgtaagctaccgtccgagtgacttcggtgcgtatctggatgcgctggaa                                                                                                                                                          <  1:277580‑M1/295‑139 (MQ=255)`  
                                                                                                                                                        `gCTCGTAAAGAGGCCACACAGTCACGCGCTCTGGCGATTCGCTCCAACGAACTGGCTGACAGTGCATCCACTAAAGTTACCGAGGCTGCCCGTGTGGCAAACCAAGCTCAACAGCTTTCCAAATTATTTGAGGAATCAAACAGGAGAAAACATTATGatcgtttctgagatagaagttaacgcactattagagagcgtcactaagttccacttcggggttatctacgactactccaccgctgagtacgtaagctaccgtccgcgcgactcaggtgcgtatctggatgcgcgtgaagccttt                                                                                                                                                    >  1:299369‑M1/1‑157 (MQ=255)`  
                                                                                                                                                         `cTCGTAAAGAGGCCACACAGTCACGCGCTCTGGCGATTCGCTCCAACGAACTGGCTGACAGTGCATCCACTAAAGTTACCGAGGCTGCCCGTGTGGAAAACCAAGCTCAACAGCTTTCCCAATTCTTTGAGTAATCAAACAGGAGAAACCATTATGatcgtttctgacatcgaagctaacgccctcttagagagcgtcactaagttccactgcggggttatctacgactactccaccgctgagtacgtaagctaccgtccgagtgacttcggtgcgtatcctgtctcttctacacatctcc                                                                                                                                                   >  1:167183‑M1/1‑156 (MQ=255)`  
                                                                                                                                                           `cGTAAAGAGGCCACACAGTCACGCGCTCTGGCGATTCGCTCCAACGAACTGGCTGACAGTGCATCCACTAAAGTTACCGAGGCTGCCCGTGTGGCAAACCAAGCTCAACAGCTTTCCAAATTCTTTGAGTAATCAAACAGGAGAAACCATTATGatcgtttctgacatcgaagctaacgccctcttagagagcgtcactaagttccacggcggggttatctacgactactccaccgctgagtacgtaagctaccgtccgagtgacttcggtgcgtatctggatgcgctggaagccgaggtt                                                                                                                                                 <  1:42449‑M1/301‑148 (MQ=255)`  
                                                                                                                                                             `tAAAGAGGCCACACAGTCACGCGCTCTGGCGATTCGCTCCAACGAACTGGCTGACAGTGCATCCACTAAAGTTACCGAGGCTGCCCGTGTGGCAAACCAAGCTCAACAGCTTTCCAAATTCTTTGAGTAATCAAACAGGAGAAACCATTATGatcgtttctgacatcgaagctaacgccctcttagagagcgtcactaagttccactgcggggttatctacgactactccaccgctgagtacgtaagctaccgtccgagtgacttcggtgcgtacctggatgcgctggaagccgaggttgc                                                                                                                                               >  1:207594‑M1/1‑152 (MQ=255)`  
                                                                                                                                                               `aaGGGGACACACAGTCACGCGCTCTGGCGATTCGCGTCAACGAACTGGCGGACAGTGCATCCACTAAAGTTACCGAGGCTGCCCGTGTGGCAAACCAAGCTCAACAGCTTTCCAAATTCTTTGAGTAATCAAACAGGAGAAACCATTATGatcgtttctgacatcgaagctaacgccctcttagagagcgtcactaagttccactgcggggttatctacgactactccaccgctgagtacgtaagcgaccgtccgagtgacttcggtgcgtatctggatgcgctggaagccgaggttgcac                                                                                                                                             <  1:46215‑M1/301‑152 (MQ=255)`  
                                                                                                                                                                              `cACTCGCTCTGGCGATTCGCTCCAACGAACTGGCTGACAGTGCATCCACTAAAGTTACCGAGGCTGCCCGTGTGGCAAACCAAGCTCAACAGCTTTCCAAATTCTTTGAGTAATCAAACAGGAGAAACCATTATGatcgtttctgacatcgaagctaacgccctcttagagagcgtcactaagttccactgcgtggttatctacgactactccaccgctgagtacgtaagctaccgtccgagtgacttcggtgcgtatctggatgcgctggaagccgaggttgcacgaggcggtcttattg                                                                                                                              <  1:269026‑M1/301‑167 (MQ=255)`  
                                                                                                                                                                              `cACGCGCTCTGGCGATTCGCTCCAACGAACTGGCTGACAGTGCATCCACTAAAGTTAACGAGGCTGCCCGTGTGGCAAACCAAGCTCAACAGCTTTCAAAATTCTTTGAGTAATCAAACAGGAGAAACCATTATGatcgtttctgacatcgaagctaacgccctcttagagagcgtcactaagttccactgcggggttatctacgactactccaccgctgagtacgtaagctaccgtccgagtgacttcggtgcgtatctggatgcgctggaagccgaggttgcacgaggcggtcttattg                                                                                                                              <  1:113875‑M1/301‑167 (MQ=255)`  
                                                                                                                                                                              `cACGCGCTCTGCCGATCCGCTGCCACGAGTTGGCTGACCGTTCATCCACTAAAGTTACCGAGGCTGCCCGTTTGTCAAACCAAGCTCAACAGCTTTCCAAATTCTTTTAGTAATCAAACAGGAGAAACCATTATGatcgtttctgacatcgaagctaacgccctcttagagagcgtcactaagttccactgcggggttatctacgactactccaccgctgagtacgtaagctaccgtccgagtgacttcggtgcgtatctggatgcgctggaagccgaggttgcacgaggcggtcttattg                                                                                                                              <  1:449094‑M1/301‑167 (MQ=255)`  
                                                                                                                                                                                  `aacactgacgATACGCTCCAACGAACGGGATGACAGTTCATCCACTAAAGTTACCGGGGCTACCCGTGTGGCAAACCAAGCACAACAGC‑TGCCAAATTCTTAGAGAAAACAAACAGGAGAAACAAGTATGaacgtgtcggatatcgaagctaacgccctattagagagaatcactaagtaccactgcggggttatctacaactactccaccgctgagtacgtaatctaccgtccgagtgacttcggtgcgtatctagatgcgcttgaagccgaggttgcacgaggcggtcttattgtgttc                                                                                                                         <  1:281662‑M1/297‑172 (MQ=255)`  
                                                                                                                                                                                    `ctctGGCGATTCGCTCCAACGAACTGGCTGACAGTGCATCCACTAAAGTTACCGAGGCTGCCCGTGTGGCAAACCAAGCTCAACAGCTTTCCAAATTCTTTGAGTAATCAAACAGGAGAAACCATTATGatcgtttctgacatcgaagctaacgccctcttagagagcgtcactaagttccactgcgtggttatctacgactactccaccgctgagtacgtaagctaccgtccgagtgacttcggtgcgtatctggatgcgctggaagccgaggttgcacgaggcggtcttattgtgttcc                                                                                                                        >  1:91467‑M1/1‑129 (MQ=255)`  
                                                                                                                                                                                                       `tataagagacagcagtgcatccactAAAGTTACCGAGGCTGCCCGTGTGGCAAACCAAGCTCAACAGCTTTCCAAATTCTTTGAGTAATCAAACAGGAGAAACCATTATGatcgtttctgacatcgaagctaacgccctcttagagagcgtcactaagttccactgcggggttatctacgactactccaccgctgagtacgtaagctaccgtccgagtgacttcggtgcgtatctggatgcgctggaagccgaggttgcacgaggcggtcttattgtgttccacaacggtcacaagtatga                                                                                                     <  1:130909‑M1/289‑192 (MQ=255)`  
                                                                                                                                                                                                        `gAACTGGCTGACAGTGCATCCACTAAAGTTACCGAGGCTGCCCGTGTGGCAAACCAAGCTAAACAGCTTTCCAAATTCTTTGAGTAATCAAACAGGAGAAACCATTATGatcgtttctgacatcgaagctaacgccctcttagagagcgtcactaagttccactgcggggttatctatgactactccaccgctgagtacgtaagctaccgtccgagtgacttcggtgcgtatctggatgcgctggaagccgaggttgaacgaggcggtcttattgctgtctcttatacacatctccgagcc                                                                                                    >  1:486759‑M1/1‑109 (MQ=255)`  
                                                                                                                                                                                                        `aaacTGGCTGACAGTGCATCCACTAAAGTTACCGAGGCTGCCCGTGTGGCAAACCAAGCTCAACAGCTTTCCAAATACTTGGAGTAATCAAACAGGAGAAAGCAATATGctcgtttttgacatcgaagctaacgccctcttagatagcgtcactaagtcccactgcggggttatctacgactactccaccgctgagtacgtaatctaccgtccgagtgacttcgatgcgtatctggatgcgctggaagccgaggttgcacgaggcggtcttattgtgttccacaacggtcacaagtatgac                                                                                                    <  1:95322‑M1/300‑193 (MQ=255)`  
                                                                                                                                                                                                         `acagggaagacagggcaTCCAGTAAAGATACCGAGGATGACCGTGTGGCAAACAAAGCTCAACAGCATTCCAAAATCATAGAGTAATCAAACAAGAGAAAACAGTATGatcgtttctgacatcgaagctaacgccctcttagagagcgtcactaagttccactgcggggttatctacgactacgccaccgctgagtacgtaagctaccgtccgagtgacttcggtgcgtatctggatgcactggaagccgaggttgcacgaggcggtcttattgtgttccacaacggtcacaagtatgaca                                                                                                   <  1:457494‑M1/293‑194 (MQ=255)`  
                                                                                                                                                                                                         `aaCTGGCTGACAGTGCATCCACTAAAGTTACCGAGGCTGCCCGTGTGGCAAACCAAGCTCAACAGCTGTCCAAATTCTTTGAGTAATCAAACAGGAGAAACCATTATGatcgtttctgacatctaagctaacgccctcttagagagcgtcactaagttccactgcggggttatctacgactactccaccgctgagtacgtaagctaccgtccgagtgacttcggtgcgtatctggatgcgctggaagccgaggttgcacgaggcggtcttattgtgttccacaacggtcacaagtatgacg                                                                                                   <  1:436813‑M1/301‑194 (MQ=255)`  
                                                                                                                                                                                                             `ggCTGACAGTGCATCCACTAAAGTTACCGAGGCTGCCCGTGTGGCAAACCAAGCTCAACAGCTTTCCAAATTCTTTGAGTAATCAAACAGGAGAAACCATTATGatcgtttctgacatcgaagctaacgccctcttagagagcgtcactaagttccactgcgtggttatctacgactactccaccgctgagtacgtaagctaccgtccgagtgacttcggtgcgtatctggatgcgctggaagccgaggttgcacgaggcggtcttattgtgttccacaacggtcacaagtatgacgttcc                                                                                               >  1:289828‑M1/1‑104 (MQ=255)`  
                                                                                                                                                                                                             `ggCTGACAGTGCATCCACTAAAGTTACCGAGGCTGCCCGTGTGGCAAACCAAGCTCAACAGCTTTCCAAATTCTTTGAGTAATCAAACAGGAGAAACCATTATGatcgtttctgacatcgaagctaacgccctcttagagagcgtcactaagttccactgcggggttatctacgactactccaccgctgagtacgtaagctaccgtccgagtgacttcggtgcgtctctggatgcgctggaagccgaggttgcacgagtcggtcttattgtgttccccaacggtcccaagtatgacgttcc                                                                                               >  1:6653‑M1/1‑104 (MQ=255)`  
                                                                                                                                                                                                               `cTGACAGTGCATCCACTAAAGTTACCGAGGCTGCCCGTGTGGCAAACCAAGCTCAACAGCTTTCCAAATTCTTTGAGTAATCAAACAGGAGAAACCATTATGatcgtttctgacatcgaagctaacgccctcttagagagcgtcactaagttccactgcggggttatctacgactactccaccgctgagtacgtaagctaccgtccgagtgacttcggtgcgtatctggatgcgctggaagccgaggttgcacgaggcggtctcattgtgttccacaacggtcacaagtatgacgttcctg                                                                                             <  1:377421‑M1/301‑200 (MQ=255)`  
                                                                                                                                                                                                                   `cAGTGCATCCACTAAAGTTACCGAGGCTGCCCGTGTGACAAACCAAGCTCAACAGCTTTCCAAATTCTTTGAGTAATCAAACAGGAGAAACCATTATGatcgtttctgacatcgcagctaacgccctcttagagagcggcactaagttccactgcggggttatctacgactactccaccgctgagtacgtaagctaccgtccgagtgacttcggtgcgtatctggatgcgctggaagccgcggttgcacgaggccgtcttattgttttccccaacggtcgcaagtatgacgttccttcatt                                                                                         >  1:101400‑M1/1‑98 (MQ=255)`  
                                                                                                                                                                                                                     `gTGCATCCACTAAAGTTACCGAGGCTGCCCGTGTGGCAAACCAAGCTCAACAGCTTTCCAAATTCTTTGAGTAATCAAACAGGAGAAATCAATATGatcgtttctgacatcgaagctaacgccctcttagagagcgtcactaagttccactgcggggttatctacgactactccaccgctgagtacgtaagctaccgtccgagtgacttcggtgcgtatctggatgcgcttgaagccgaggttgcacgaggcggtcttattgtgttccacaacggtcacaagtatgacgttcctgcattga                                                                                       >  1:480921‑M1/1‑96 (MQ=255)`  
                                                                                                                                                                                                                       `gCATCCACTAAAGTTACCGAGGCTTCCCGGGTGGCAAACCAAGCTCAACAGCTTTCCAAATTCTTTGAGTAATCAAACAGGAGAAACCATTATGatcgtttctgacatcgaagctaacgccctcttagagagcgtcactaagttccactgcggggttatctacgactactccaccgctgagtacgtaagctaccgtccgagtgactgcggtgcgtatctggatgcgctggaagccgaggttgcacgaggcggtcttattgtgttccacaacggtcacaagtatgacgttcctgcattgacc                                                                                     <  1:396546‑M1/301‑208 (MQ=255)`  
                                                                                                                                                                                                                           `ccACTAAAGTTACCGAGGCTGCCCGTGTGGCAAACCAAGCTCAACAGCTTTCCAAATTCTTTGAGTAATCAAACAGGAGAAACCATTATGatcgtttctgacatcgaagctaacgccctcttagagagcgtcactaagttccactgcggggttatctacgactactccaccgctgagtacgtaagctaccgtccgagtgacttcggtgcgtatctggatgcgctggaagccgaggttgcacgaggcggtcttattgtgttccacaacggtcacaagtatgacgttcctgcattgaccaaac                                                                                 >  1:7248‑M1/1‑90 (MQ=255)`  
                                                                                                                                                                                                                                  `aGTTACCGAGGCTGCCCGTGTGGCAAACCAAGCTCAATAGCTTTCCAAATTCTTTGAGTAATCAAACAGGAGAAACCATTATGatcgtttctgacatcgaagctaacgccctcttagagagcgtcactaagttccactgcggggttatctacgactactccaccgctgagtacgtaagctaccgtccgagtgacttcggtgcgtatctggatgagctggaagcctaggttgcacgaggcggtcttattgtgttccacaacggtcacaagtatgacgttcctgcattgaccaaactggcaaa                                                                          <  1:179200‑M1/301‑219 (MQ=255)`  
                                                                                                                                                                                                                                  `aGTTACCGAGGCTGCCCGTGTGGCAAACCAAGCTCAACAGCTTTCCAAATTCTTTGAGTAATCAAACAGGAGAAACCATTATGatcgtttctgacatcgaagctaacgccctcttagagagcgtcactaagttccactgcggggttatctacgactactccaccgctgagtacgtaagctaccgtccgagtgacttcggtgcgtatctggatgcgctggaagccgaggttgcacgaggcggtcttattgtgttccacaacggtcacaagtatgacgttcctgcattgaccaaactggcaaa                                                                          >  1:373868‑M1/1‑83 (MQ=255)`  
                                                                                                                                                                                                                                    `ttACTGAGGCTGCCCGTGTGGCAAACCAAGCTCAACAGCTTTCCAAATTCTTTGAGTAATCAAACAGGAGAAACCATTATGatcgtttctgacatcgaagctaacgccctcttagagagcgtcactaagttccactgcgggtttatctacgactactccaccgctgagtacgtaagctaccgtccgagtgacttcggtgcgtatctggatgcgctggaaaccgtggttgcacgaggcggtcttattgtgttcctcaacggtcacaagtatgacgttcctgcattgaccaaactggtaaaat                                                                        >  1:247998‑M1/1‑81 (MQ=255)`  
                                                                                                                                                                                                                                          `aGGCTGCCCGTGTTGCAAAGCAAGCTCAACAGCTTTCCAAATTCTTTGAGTAATCAAACAGGAGAAACCAGTATGatcgtttctgacatcgaagctaacgccctcttagatggcgtcactaagttccactgcggggttatctacgactactccaccgctgagtacgtaagctaccgttcgagtgacttcggtgcgtatctggatgcgctggaagccgaggttgcacgaggcggtcttattgtgttccacaacggtcacaagtatgacgttcctgcattgaccaaactggcaaagttgcaat                                                                  <  1:139340‑M1/301‑227 (MQ=255)`  
                                                                                                                                                                                                                                                 `ccGTGTGGCAAACCAAGCTCAACAGCTTTCCAAATTCTTTGAGTAATCAAACAGGAGAAACCATTATGatcgtttctgacatcgaagctaacgccctcttagagagcgtcactaagttccactgcggggttatctacgactactccaccgctgagtacgtaagctaccgtccgagtgacttcggtgcgtatctggatgcgctggaagccgaggttgcacgaggcggtcttattgtgttccacaacggtcacaagtatgacgttcctgcattgaccaaacttgcaaagttgcaattgaaccg                                                           >  1:433367‑M1/1‑68 (MQ=255)`  
                                                                                                                                                                                                                                                   `gtgtGGCAAACCAAGCTCAACAGCTTTCCAAATTCTTTGAGTAATCAAACAGGAGAAACCATTATGatcgtttctgacatcgaagctaacgccctcttagagagcgtcactaagttccactgcggggttatctacgactactccaccgctgagtacgtaagctaccgtccgagtgacttcggtgcgtatctggatgcgctggaagccgaggttgcacgaggcggtcttattgtgttccacaacggtcacaagtatgacgttcctgcattgaccaaactggcaaagttgcaattgaaccgag                                                         <  1:311525‑M1/301‑236 (MQ=255)`  
                                                                                                                                                                                                                                                      `gtgggctaggatatgtgtataagagacagttctttgagtaatcaaacaggagaaactattATGatcgtttctgacatcgcagctagcgccctcttagagagcgtcactaagttccactgcggggttatctacgacttctccaccgctgattacgtaagctaccgtccgagtgacttcggtgcgtatctggatgcgctggaagccgaggttgcacgaggcggtcttattgtgttccacaacggtcacaagtatgacgttcctgcattgaccaaactggcaaagttgcaattgaaccgagagt                                                      <  1:382934‑M1/272‑239 (MQ=255)`  
                                                                                                                                                                                                                                                             `gtgtataagatacagttccaaattctttgagtAATCAAACAGGAGAAACCATTATGatcgtttctgacatcgaagctaacgccctctgagagagcgtcactaagttccactgcggggttatctacgactactccaccgctgagtacgtaagctaccgtccgagtgacttcggtgcgtatctggatgcgctggaagccgaggttgcacgaggcggtcttattgtgttccacaacggtcacaagtatgacgttcctgcattgaccaaactggcaaagttgcaattgaaccgagagttccacct                                               <  1:255643‑M1/286‑246 (MQ=255)`  
                                                                                                                                                                                                                                                               `aaGCTCACTATCTTTCCAAATTCTTTGAGTAATCACACAGTAGAAATCAATATGatcgtttctgacatcgaagctaacgccctcttagagagcgtcactaagttccactgcgtggttatctacgactactccaccgctgagtacgtaagctaccgtccgagtgacttcggtgcgtatctggattcgctggaagccgaggttgcacgaggcggtcttattgtgttccacaacggtcacaagtatgacgttcctgcattgaccaaactggcaaagttgcaattgaaccgagagttccaccttc                                             <  1:126490‑M1/301‑248 (MQ=255)`  
                                                                                                                                                                                                                                                                 `gCTCAACAGCTTTCCAAATTCTTTGAGTAATCAAACAGGAGAAACCATTATGatcgtttctgacatcgaagctaacgccctcttagagagcgtcactaagttccacttcggggttatctacgactactccaccgctgagtacgtaagctaccgtccgagtgacatcggtgcgtatctggttgctctggaagccgaggttgcacgaggcggtcttattgtgttccacaacggtcccaagtatgacgttcctgcattgaccaaactggcaaagttgcaattgaaccgagagttccaccttccc                                           >  1:400740‑M1/1‑52 (MQ=255)`  
                                                                                                                                                                                                                                                                  `atttgtataagttactgttctttgagtaatcaaacaGGAGAAACCATTATGatcgtttctgacatcgaagctaacgccctcttagagatcgtcactaagttccactgctgggttatctacgactactccaccgctgagtacgtaagctaccgtccgagtgacttcggtgcgtatctggatgcgctggaagccgaggttgcacgaggcggtcttattgtgttccacaacggtcacaagtatgacgttcctgcattgaccaaactggcaaagttgcaattgaaccgagagttccaccttcctc                                          <  1:109918‑M1/284‑251 (MQ=255)`  
                                                                                                                                                                                                                                                                                  `aTTCTTTGAGTAATCAAACAGGAGAAACCATTATGatcgtttctgacatcgaagctaacgccctcttagagagcgtcactaagttccactgcggggttatctacgactactccaccgctgagtacgtaagctaccgtccgagtgacttcggtgcgtatctggatgcgctggaaaccgaggttgcacgaggcggtcttattgtgttccacaacggtcacaagtatgacgttcctgcattgaccaaactggcaaagttgcaattgaaccgagagttccaccttcctcgtgagaactgtattga                          <  1:316042‑M1/301‑267 (MQ=255)`  
                                                                                                                                                                                                                                                                                   `ttCTTTGAGTAAACAAAAATGAGAAACCATTATGagcgtttctgacatcgaagctaacgcccgcttagagagagtaactaagttccactgcggggttatctacgactactccaacgctgagtacgtaagctaccgtccgagtgacttcggtgcgtatctggatgcgctggaagccgaggttgcacgaggcggtcttattgtgttccacaacggtcacaagtatgacgttcctgcattgaccaaactggcaaagttgcaattgaaccgagagttccaccttcctcgtgataactgtattgac                         <  1:33336‑M1/301‑268 (MQ=255)`  
                                                                                                                                                                                                                                                                                             `agacagctgggagaaaccatTATGatcgtttctgaaatcgaagctaacgccctcttagagagcgtcactaagttccactgcggggttatctacgactactccaccgatgagtacgtaagctaccgtccgagtgacttcggtgcgtatctggatgcgctggaagccgaggttgcacgaggcggtcttattgtgttccacaacggtcacaagtatgacgttcctgcattgaccaaaccggcaaagttgcaattgaaccgagagttccaccttcctcgtgagaactgtattgacacccttgtgt               <  1:493244‑M1/292‑278 (MQ=255)`  
                                                                                                                                                                                                                                                                                             `aaTCAAACAGGAGAAACCATTATGatcgtttctgacatcgaagctaacgccctcttagagagcgtcactaagttccactgcggggttatctacgactactccaccgctgagtacgtaagctaccgtccgagtgacttcggtgcgtatctggatgcgctggaagccgaggttgcacgaggcggtcttattgtgttccacaacggtcacaagtatgacgttcctgcattgaccaaactggcaaagttgcaattgaaccgagagttccaccttcctcgtgagcactgtattgacaaccttgtgt               >  1:210679‑M1/1‑24 (MQ=255)`  
                                                                                                                                                                                                                                                                                                  `aaCAGGAGAAACCATTATTatcgtttctgacatcgaagctaacgccctcttagagagcgtcactaagttccactgcggggttatctacgactactccaccgctgagtacgtaagctaccgtccgagtgacttcggtgcgtatctggatgcgctggaagccgaggttgcacgaggcggtcttattgtgttccacaacggtcacaagtatgacgttcctgcattgaccaaactggcaaagttgtaattgaaccgagagttccaccttcctcgtgagaactgtattgacacccttgtgttgtca          <  1:25343‑M1/301‑283 (MQ=255)`  
                                                                                                                                                                                                                                                                                                  `aaCAGGAGAAACCATTATGatcgtttctgacatcgaagctaacgccttcttagagagcgtcactaagttccactgcggggttatctacgactactccaccgctgagtacgtaagctaccgtccgagtgacttcggtgcgtatctggatgcgctggaagccgaggttgcacgaggcggtcttattgtgttccacaacggtcacaagtatgacgttcctgcattgaccaaactggcaaagtttcaattgaaccgagagttccaccttcctcgtgagaactgtattgacacccttgtgttgtca          <  1:204126‑M1/301‑283 (MQ=255)`  
                                                                                                                                                                                                                                                                                                      `ggAGAAACCATTATGatcgtttctgatatcgaagctaacgccctcttagagagcgtcactaagttccactgcggggttatttacgactactccaccgctgagtacgtaagctaccgtccgagtgacttcggtgcgtatctggatgcgctggaagccgaggttgcacgaggcggtcttattgtgttccacaacggtcacaagtatgacgttcctgcattgaccaaactgtcaaagttgcaattgaaccgcgagttccaccttcctcgtgagaactttattgacacccttgtgttataccgct      >  1:360448‑M1/1‑15 (MQ=255)`  
                                                                                                                                                                                                                                                                                                          `aaaaCATTATGatcgtttctgcaatcgaagctaacgccctcttagagagcgtcactaagttccactgcggggttatctacgactactccaccgctgagtacgtaagctaccgtccgcgtgacttcggtgcgtatctggatgcgctggaagccgaggttgcacgaggcggtcttattgtgttccacaacggtcacaagtatgacgttcctgcattgaccaaactggcaaagttgcaattgaaccgagagttccaccttcctcgtgagaactgtattgacacccttgtgttgtcacgtttgat  <  1:322259‑M1/301‑291 (MQ=255)`  

                                                                                                                                                                                                                                                                                                            `|`                                                                                                                                                                                                                                                                                                            
`TGACTTTCCAGACGACTACGAGAAGTTTCGCTGGAGAGTCCCATTCTAATACGACTCACTAAAGGAGACACACCATGTTCAAACTGATTAAGAAGTTAGGCCAACTGCTGGTTCGTATGTACAACGTGGAAGCCAAGCGACTGAACGATGAGGCTCGTAAAGAGGCCACACAGTCACGCGCTCTGGCGATTCGCTCCAACGAACTGGCTGACAGTGCATCCACTAAAGTTACCGAGGCTGCCCGTGTGGCAAACCAAGCTCAACAGCTTTCCAAATTCTTTGAGTAATCAAACAGGAGAAACCATTATGTCTAACGTAGCTGAAACTATCCGTCTATCCGATACAGCTGACCAGTGGAACCGTCGAGTCCACATCAACGTTCGCAACGGTAAGGCGACTATGGTTTACCGCTGGAAGGACTCTAAGTCCTCTAAGAATCACACTCAGCGTATGACGTTGACAGATGAGCAAGCACTGCGTCTGGTCAATGCGCTTACCAAAGCTGCCGTGACAGCAATTCATGAAGCTGGTCGCGTCAATGAAGCTATGGCTATCCTCGACAAGATTGATAACTAAGAGTGGTATCCTCAAGGTCGCCA  >  NC_001604.1/13278‑13876`

  

Alignment Legend

Aligned base mismatch/match (shaded by quality score): `ATCG/ATCG` `< 3 ≤` `ATCG/ATCG` `< 9 ≤` `ATCG/ATCG` `< 18 ≤` `ATCG/ATCG` `< 32 ≤` `ATCG/ATCG` `< 38 ≤` `ATCG/ATCG`

Unaligned base: `atcg`    Masked matching base: `atcg`    Alignment gap: `‑`    Deleted base: `‑`

Reads not counted as support for junction

read\_name Not counted due to insufficient overlap past the breakpoint.

read\_name Not counted due to not crossing MOB target site duplication.
